# Longitudinal changes in occupational well-being: a four-wave panel survey of employees in Switzerland and Germany

**DOI:** 10.1186/s12889-025-25237-z

**Published:** 2025-11-28

**Authors:** Martin Tušl, Anja I. Lehmann, Anja I. Morstatt, Holger Dressel, Georg F. Bauer

**Affiliations:** 1 Public and Organizational Health, Center of Salutogenesis, Institute of Epidemiology, Biostatistics, and Prevention, University of Zurich, Zurich, Switzerland; 2 Work, Organizational and Social Psychology, Institute of Psychology, Technische Universität Braunschweig, 38106 Braunschweig, Germany; 3 Occupational and Environmental Medicine, Institute of Epidemiology, Biostatistics, and Prevention, University of Zurich, Zurich, Switzerland

**Keywords:** Work engagement, Exhaustion, Latent change score modeling, Longitudinal study, COVID-19 pandemic

## Abstract

**Background:**

Occupational well-being is a key element of employee health and a crucial determinant of productivity. The present exploratory study investigates longitudinal changes in work engagement and exhaustion as indicators of occupational well-being during different stages of the COVID-19 crisis among employees in Switzerland and Germany.

**Methods:**

Using a four-wave panel design, we collected data from 2,137 participants during various stages of the pandemic from April 2020 to December 2021. We applied latent change score modeling to examine changes within groups and differences between employee groups over time.

**Results:**

Results show that work engagement declined significantly between April and July 2020, partially recovered between July and December 2020, and then remained stable until December 2021. Multi-group analysis highlighted the importance of national context, social connections, and work flexibility as working in Switzerland, living with others, caregiving responsibilities for children, and remote work were consistently associated with higher work engagement. In contrast, exhaustion levels remained relatively stable across all time points. However, some significant differences emerged between groups, with employees in Switzerland reporting an overall lower level of exhaustion. Age-specific trends were also identified, with older employees reporting overall lower levels of exhaustion compared to younger employees across the whole measurement period.

**Conclusion:**

The study contributes to the understanding of the dynamic nature of occupational well-being during the pandemic, with implications for organizations aiming to support their employees not only in times of crises.

**Supplementary Information:**

The online version contains supplementary material available at 10.1186/s12889-025-25237-z.

## Background

The COVID-19 pandemic has profoundly transformed work environments, leading to significant disruptions and adaptations. These transformations introduced new challenges and opportunities, which have underscored the need to understand how occupational well-being fluctuates over time in response to such disruptions. Occupational well-being is a key element of employee health and a crucial determinant of productivity [1, 2]. Work engagement and exhaustion are commonly used as its indicators [3, 4]. Work engagement is defined as a positive, fulfilling, work-related state of mind characterized by vigor, dedication, and absorption in one’s work [5]. In contrast, exhaustion is a core symptom of occupational burnout defined as a negative state of physical, emotional, and mental exhaustion resulting from chronic stress in the workplace [6]. The two constructs emerge from distinct but interrelated processes, as conceptualized by the Job Demands-Resources model (JD-R) [4, 7]. Work engagement results from a motivational process in resource-rich environments that fosters positive outcomes, both for employees and organizations. Occupational burnout, on the other hand, reflects a health-impairing process typical of highly demanding and resource-depleted work environments, leading to negative health and performance outcomes [8, 9].

Occupational well-being is generally more variable than stable, as shown in studies conducted both before [10, 11] and during the pandemic [12, 13]. This variability is shaped by a range of sociodemographic factors and working conditions. For example, younger employees often experience greater fluctuations in well-being compared to older employees, and they were also strongly affected by pandemic-related stressors such as increase in job insecurity and social isolation [14, 15]. Gender differences also play a critical role, with female employees reporting significant declines in well-being during the pandemic, particularly in terms of higher exhaustion levels [16]. Similarly, employees with lower education levels are more likely to have jobs characterized by fewer resources and higher job insecurity, which makes them more vulnerable in times of crisis [17, 18]. Living situations is another key factor for occupational well-being especially during the periods of imposed social restrictions, which exacerbate feelings of isolation for those living alone [19, 20]. Living in a family household generally serves as a protective factor for mental health [21]. However, caregiving responsibilities, especially for underage children, can exacerbate work-family conflict, potentially undermining occupational well-being [22, 23]. Finally, the pandemic triggered a widespread shift to remote work [16]. While some studies indicate that employees generally viewed this transition positively [13, 24, 25], its effects may have not been uniform. Employees with prior remote work experience may have adapted more easily, whereas those new to remote work faced unique challenges in adjusting to the forced change. Therefore, the long-term impact of this shift on occupational well-being, particularly in relation to differing levels of remote work experience, remains unclear [26, 27].

As contextual factors, such as the pandemic, profoundly and differentially shape employee experiences and behaviors [28], understanding how occupational well-being varies among different groups of employees during crises strengthens our knowledge of group-specific responses to changing work environments [29]. This insight is necessary for developing targeted workplace interventions that effectively promote occupational well-being among those most at risk during future crises [30]. While prior research has documented some of these patterns [12, 13, 24], the long-term impact of the pandemic on group-specific occupational well-being remains underexplored.

To address this gap, our exploratory study aims to answer the following research questions: (1) How did work engagement and exhaustion as key indicators of occupational well-being evolve longitudinally across different stages of the COVID-19 pandemic?; (2) How did sociodemographic factors and work characteristics (e.g., age, gender, education, living situation, caregiving responsibilities, remote work) influence changes in work engagement and exhaustion? By investigating these research questions, our study aims to identify group-specific vulnerabilities and resilience factors, to inform targeted interventions that support employee well-being during future crises.

The study targets employees in Germany and the German-speaking part of Switzerland during the COVID-19 pandemic. These two countries were selected due to their geographic proximity and economic comparability, yet they differ in certain structural and cultural aspects relevant to the workplace. Moreover, both countries implemented comparable restrictions in response to the pandemic, although Switzerland generally maintained slightly less stringent measures [31].

## Methods

### Design

The study is based on an online survey with a four-wave panel design conducted from April 2020 to December 2021, capturing different stages of the COVID-19 pandemic. The first wave of data collection (T1) occurred in April 2020 during the initial stage of the pandemic and the first lockdown. The second wave (T2) occurred at the end of June 2020 and beginning of July 2020 during the relaxation of lockdown measures. The third wave (T3) was conducted in December 2020 during reintroduction of restrictive measures, and the fourth wave took place a year later towards the end of the acute phase of the COVID-19 pandemic in December 2021. The intervals between waves were chosen based on the evolving context of the COVID-19 pandemic and general recommendations regarding the optimal time lags necessary to detect changes in the studied variables [32]. The data used in the present study were partly collected within the larger project *Craft4Health*^1^. The specific part of the questionnaire relevant to this study is provided as supplementary material.

### Participants

The study targeted the general working population in Germany and the German-speaking region of Switzerland. We included participants who were employed, working more than 20 h per week, and were within the age range of 18 to 65 years. Data collection was facilitated by a professional panel data service using quota sampling to recruit a sample that is a good representation of the general working population in Germany and Switzerland. Data were collected via an online questionnaire using a web-based survey provider. Participation was voluntary and participants received a small incentive for completing the survey in the form of redeemable points. Informed consent was obtained at the beginning of the study. Each participant in the panel service database was assigned a unique code to ensure anonymity and prevent multiple submissions. Survey items were mandatory and participants could not advance through the questionnaire without answering all questions. To ensure data quality, several disqualifying items were added to exclude participants who likely respond carelessly (e.g., “Please choose number three as an answer to this item”). Approximately 3000 participants were invited to participate in the study, and the final sample included 2,137 participants. Of those, 2,113 completed the questionnaire at T1, 1,592 completed T2, 1,163 completed T3, and 922 completed T4 (i.e., 44% completion rate T1-T4). In total 754 participants completed the questionnaire at all four data collection points. We investigated attrition in our sample and compared those with complete participation (*N* = 754) against those with incomplete participation (*N* = 1,383, missing at least one wave), regarding main sociodemographic characteristics (age, gender, country), work engagement and exhaustion at T1. We found significant mean differences for age *M*_complete_ = 48.4 years vs. *M*_incomplete_ = 46 years (*t*(1778) = 4.898, *p* < 0.001, *d* = 0.21). A chi-square test indicated a significant association between attrition status and gender (χ²(1, *N* = 2157) = 7.31, *p* = 0.007, φ = 0.06). Females showed higher attrition (67.4% missing at least one wave) compared to males (61.6%). A chi-square test also revealed a significant association between attrition status and country (χ²(1, *N* = 2157) = 31.11, *p* = < 0.001, φ = 0.12). Swiss employees showed higher attrition (74.8% missing at least one wave) compared to German employees (61%). Effect sizes for these comparisons were small or marginal. We did not find significant mean differences for work engagement *M*_complete_ = 3.20 vs. *M*_incomplete_ = 3.32 (*t*(1501) = 1.93, *p* = 0.054), nor for exhaustion *M*_complete_ = 2.35 vs. *M*_incomplete_ = 2.40 (*t*(1550) = 1.32, *p* = 0.19). In Table 1, we present the distribution of the full sample (*N* = 2,137) across the studied groups of employees. The gender distribution of our sample is comparable to the general working population in Germany (53% male) and Switzerland (53.3% male). However, our sample is somewhat older than the general working population in Germany (M = 43 years) and Switzerland (M = 42.3 years).

**Table 1:** Distribution of the sample

		*N*	%
Full sample		2137	100
Country			
	Germany	1613	75.5
	Switzerland	497	23.3
	n/a	27	1.3
Gender			
	Male	1141	53.4
	Female	995	46.6
	n/a	1	0.05
Age			
	18–35	448	21.0
	36–45	472	22.1
	46–55	643	30.1
	56+	574	26.9
	n/a	0	0
Education			
	Low	620	29.0
	Intermediate	392	18.3
	High	298	13.9
	n/a	827	38.7
Caring duties			
	No	1688	79.0
	Yes	449	21.0
	n/a	0	0
Living situation			
	Alone	573	26.8
	Not alone	1470	68.8
	n/a	94	4.4
Remote work			
	None	924	43.2
	Experienced	732	34.3
	New	453	21.2
	n/a	28	1.3

Note: n/a = missing data or data not included in the analyses due to changes in participants’ situation across the examined period

### Measures

*Work engagement*. We used the nine-item version of the Utrecht Work Engagement Scale [33]. This scale measures the three underlying dimensions of work engagement: vigor (e.g., “At my work, I feel bursting with energy”), dedication (e.g., “I am enthusiastic about my job”), and absorption (e.g., “I am immersed in my work”). The answering format ranges from 0 = *never* to 6 = *always*. The nine items showed excellent internal consistency in our data (α = 0.97). For the data analysis we modelled work engagement using three parcels, each computed as the mean score of the three items representing one of the subdimensions (i.e., vigor, dedication, absorption) to reduce model complexity [34]. This parceling approach retained high reliability of the measure (α = 0.96).

*Exhaustion*. We used three items from the work-related exhaustion subscale of the Copenhagen Burnout Inventory [3], which capture the extent of physical and psychological exhaustion attributed to work (e.g., “Do you feel worn out at the end of the working day?”; “Are you exhausted in the morning at the thought of another day at work?”; “Do you feel that every working hour is tiring for you?”). Items were rated on a 5-point scale from 1 = *never/almost never* to 5 = *very often*. Internal consistency was adequate (α = 0.83).

In addition, we repeatedly collected data on several key sociodemographic and work characteristics as grouping variables to examine how these diverse factors influence occupational well-being.

*Country*. To account for contextual differences, we included country as a variable and coded it as 1 = *Germany* and 2 = *Switzerland*, *N* = 27 participants were excluded from the analyses as they did not belong to either group.

*Gender and age*. Gender was coded as 1 = *Male* and 2 = *Female*, *N* = 1 participant was excluded from the analyses as they did not belong to either group. Age was coded into four categories to reflect different life stages, approximating with Levinson’s theory of adult development [35]: 1 = *18–35 years (early adulthood)*, 2 = *36–45 years (transition to middle adulthood)*, 3 = *46–55 years (middle adulthood)*, 4 = *More than 55 years (culminating middle adulthood)*.

*Education.* Education level was coded into three categories based on the International Standard Classification of Education (ISCED [36]: 1 = *Low* (compulsory, vocational, or upper secondary education, ISCED levels 0–3), 2 = *Intermediate* (post-secondary non-tertiary or applied sciences education, ISCED levels 4–5), and 3 = *High* (university-level education – bachelor’s, master’s, or doctoral degrees, ISCED levels 6–8). In total, *N* = 827 were coded as missing as the data on education were collected only at T3 leading to missing data for participants that dropped out between T1 and T3.

*Living situation.* We assessed employee living situation based on two categories as 0 = *Living alone* and 1 = *Not living alone* at all four data collection points. In total, *N* = 94 were excluded from the analyses due to changes in participants’ living situation across the examined period.

*Caring duties for children.* We assessed caring duties with a single item “Do you have children under 18 years who live in your household and require your care?”. We categorized the answers as 1 = *No* and 2 = *Yes*.

*Remote work.* We assessed remote work based on the percentage of remote working time from 0% to 100%. We collected data on the amount of remote work before the pandemic and since the pandemic to identify employees who had experience with remote work prior to the pandemic and those for whom it was a new experience. Employees who reported 0% remote work at all four time points were given a value of 1 = *None*, those who worked remotely at least some of their working time and had prior experience with remote work were given a value of 2 = *Experienced*, and those who worked remotely at least some of their working time during the pandemic but had no prior experience were given a value of 3 = *New.* In total *N* = 28 participants were excluded from the analyses due to changes in remote work across the examined period.

### Statistical analysis

Data analysis was carried out in R [37] with the following packages: *tidyverse* [38], and *lubridate* [39] for data handling, *psych* [40] and *lavaan* [41] for data analysis, *ggplot2* [38] and *patchwork* [42] for plot creation. To examine changes in occupational well-being across four data collection points, we applied univariate latent change score modeling (LCSM) [43], which captures both within-group change and between-group difference in change. Consistent with the JD-R model, which conceptualizes work engagement and exhaustion as distinct processes, we fitted separate models for each variable [4, 7]. Analyses were conducted using the *sem* function from the *lavaan* package [41] with the full information maximum likelihood estimation method to handle missing data from participant dropout [44]. LCSM enabled us to model change trajectories across groups of employees and to test both the significance of mean changes within groups and differences in change between groups. Since latent change scores are based on latent variables estimated from multiple observed items, they account for measurement error, providing more precise estimates of true changes and the influence of predictors on these changes than conventional statistical methods [45].

To examine differences over time and across groups, it is necessary to establish measurement invariance, which ensures that the constructs being studied maintain their structural stability (configural invariance), factor loadings (metric invariance), and intercepts (scalar invariance) across time points and/or groups [46, 47]. Establishing measurement invariance allows for meaningful comparisons by confirming that the construct’s interpretation remains consistent across groups or time. We assessed longitudinal measurement invariance for the entire sample and for subgroups (e.g., gender, age, education, etc.). Additionally, we tested group-specific invariance at T1 (e.g., comparing male vs. female or different age groups). Although measurement invariance could be assessed for the interactions between time and groups, such models tend to be very complex. We propose that our approach offers a more parsimonious alternative which still provides useful approximations of measurement invariance. Confirmatory factor analyses (CFAs) were used to evaluate the stages of measurement invariance (configural, metric, scalar), and model fit was assessed using established cut-off criteria: Root Mean Square Error of Approximation (RMSEA ≤ 0.06), Comparative Fit Index (CFI ≥ 0.95), Tucker-Lewis Index (TLI ≥ 0.95), and Standardized Root Mean Square Residual (SRMR ≤ 0.08) [48]. Model comparisons were performed using chi-square difference tests. The R code used for these analyses is provided in the supplementary materials.

### Measurement invariance

Since we used three items for both latent constructs, the configural model is just-identified so it cannot be evaluated using global fit indices on its own. However, it still provides a valid baseline for nested model comparisons. Both the metric and scalar models are overidentified, which allowed us to assess measurement invariance through Δχ², ΔCFI, and ΔRMSEA. Following established guidelines [49], we interpreted the small changes in fit as support for approximate invariance across time points. We established full scalar longitudinal measurement invariance for work engagement and partial scalar invariance for exhaustion both for the full sample and for the different groups. Looking into the separate group differences, we established full scalar measurement invariance for all groups except for gender, age, and living situation, where only partial scalar measurement invariance was obtained for work engagement. Additionally, partial scalar measurement invariance was established for exhaustion when splitting the sample by gender, country, and age. This indicates that the measures are not entirely equivalent across these groups as indicated by the need to release one intercept in both measures. Allowing for partial measurement invariance provides only an approximation, which needs to be considered for the interpretation of the results, and we refer to it in the section on limitations of our study [47]. Detailed results of the longitudinal and group measurement invariance analyses are provided in supplementary material^3^.

## Results

**Fig. 1:**
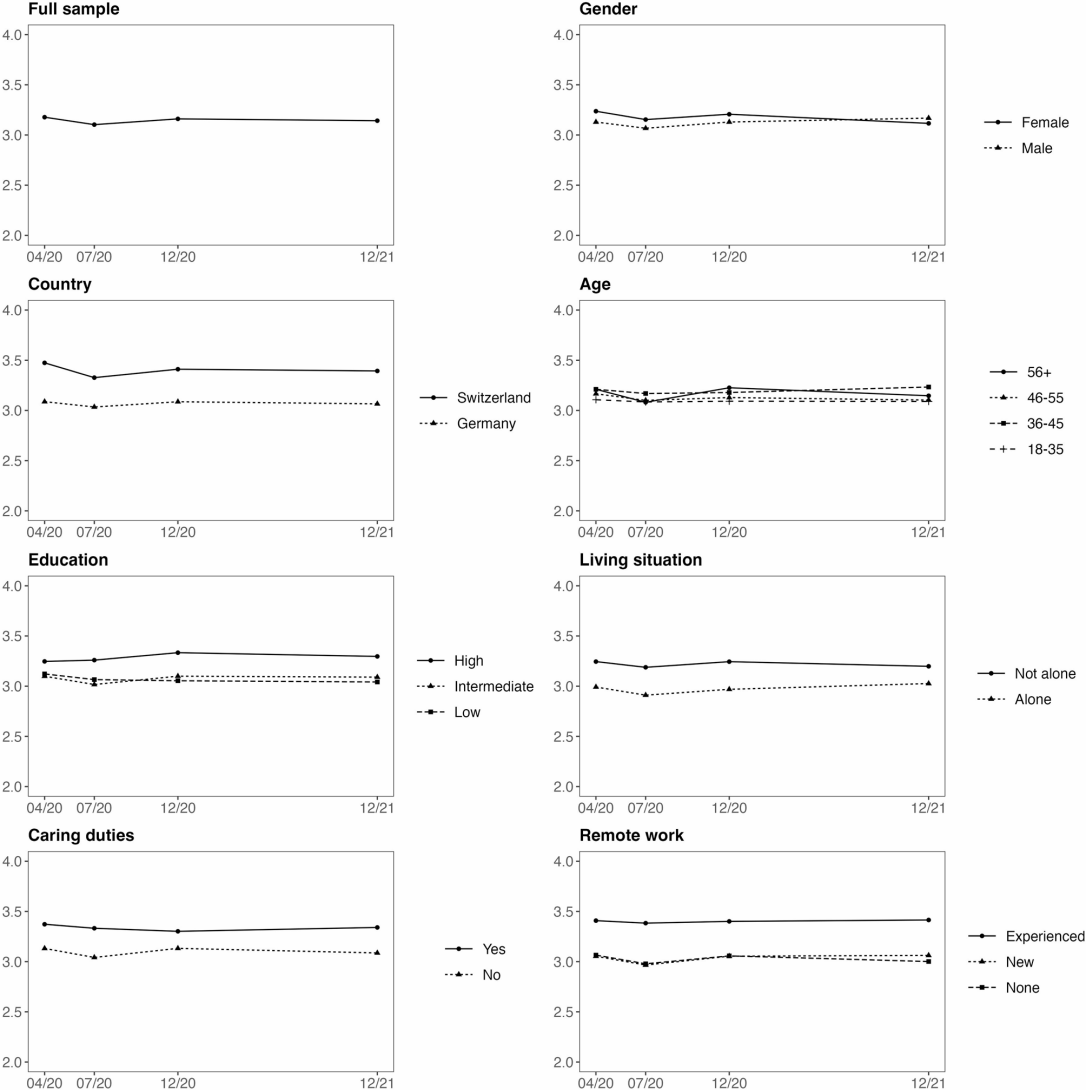
Latent change score trajectories of work engagement for different employee groups across the four measurement points from April 2020 to December 2021. The full scale for work engagement ranged from 0 to 6. For visual clarity, the y‑axis is restricted to the range 2 to 4, which covers the observed values and highlights the changes over time.

**Fig. 2:**
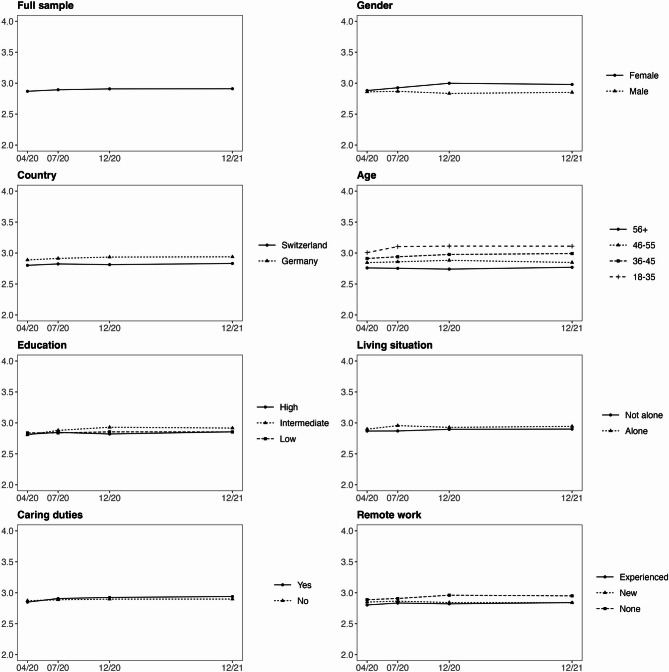
Latent change score trajectories of exhaustion for different employee groups across the four measurement points from April 2020 to December 2021. The full scale for exhaustion ranged from 1 to 5. For visual clarity, the y‑axis is restricted to the range 2 to 4, which covers the observed values and highlights the changes over time.

The results are presented in three steps. We first summarize overall trends in work engagement and exhaustion, followed by between-group comparisons, and within-group changes. In Table 2, we present intercepts at T1 (µ) as well as latent change scores (Δµ) at T2, T3 and T4 showing how work engagement and exhaustion developed over time for the full sample and for different groups of employees. Table 2 shows within group intercepts and change scores as well as between-group differences in the intercept at T1 (Δµ) and the slopes (Δβ) from T1 to T2, T2 to T3, and T3 to T4. Figures 1 and 2 show the estimated latent trajectories of work engagement and exhaustion for different groups. Detailed results including variances and standard errors are provided in supplementary material^4^.

**Table 2:** Intercepts and latent change scores in work engagement and exhaustion for the full sample and across different groups of employees including group comparisons.

	Intercepts and change scores							Group comparisons			
	µT1	βT1-T2	µT2	βT2-T3	µT3	βT3-T4	µT4	ΔµT1	ΔβT1-T2	ΔβT2-T3	ΔβT3-T4
Work engagement											
Full sample	3.177	−0.074***	3.103	0.057**	3.160	−0.018	3.142	**-**	**-**	**-**	**-**
Country											
Germany	3.088	−0.053*	3.035	0.052*	3.087	−0.021	3.066	*Ref.*			
Switzerland	3.474	−0.147***	3.327	0.084	3.411	−0.017	3.394	0.386***	−0.094*	0.032	0.004
Gender											
Male	3.129	−0.062*	3.067	0.062*	3.129	0.039	3.168	*Ref.*			
Female	3.237	−0.083**	3.154	0.053	3.207	−0.091*	3.116	0.108	−0.021	−0.009	−0.130*
Age											
18–35	3.145	−0.048	3.097	0.032	3.129	−0.030	3.099	*Ref.*			
36–45	3.218	−0.038	3.180	0.011	3.191	0.053	3.244	0.073	0.010	−0.021	0.083
46–55	3.197	−0.073*	3.124	0.017	3.141	−0.037	3.104	0.052	−0.025	−0.015	−0.007
56+	3.216	−0.133***	3.083	0.145***	3.228	−0.073	3.155	0.071	−0.085	0.113	−0.043
Education											
Low	3.099	−0.082*	3.017	0.083*	3.100	−0.010	3.090	*Ref.*			
Intermediate	3.122	−0.056	3.066	−0.011	3.055	−0.012	3.043	0.023	0.026	−0.094	−0.002
High	3.247	0.013	3.260	0.074	3.334	−0.037	3.297	0.148	0.095	−0.009	−0.027
Living situation											
Alone	2.992	−0.081*	2.911	0.058	2.969	0.057	3.026	*Ref.*			
Not alone	3.245	**−0.057**	3.188	0.056*	3.244	−0.046	3.198	0.253***	0.024	−0.002	−0.103
Caring duties											
No	3.132	−0.090**	3.042	0.091**	3.133	−0.046	3.087	*Ref.*			
Yes	3.372	−0.040*	3.332	−0.030	3.302	0.039	3.341	0.240***	0.050	−0.121	0.085
Remote work											
None	3.057	−0.087**	2.970	0.078*	3.048	−0.055	2.993	*Ref.*			
Experienced	3.405	−0.041	3.364	0.015	3.379	0.007	3.386	0.348***	0.046	−0.063	0.062
New	3.026	−0.077*	2.949	0.089	3.038	0.017	3.055	−0.031	0.010	0.011	0.072
Exhaustion											
Full sample	2.869	0.024	2.893	0.014	2.907	0.002	2.909	-	-	-	-
Country											
Germany	2.888	0.025	2.913	0.022	2.935	0.004	2.939	*Ref.*			
Switzerland	2.802	0.022	2.824	−0.012	2.812	0.019	2.831	−0.086*	−0.003	−0.034	0.015
Gender											
Male	2.859	0.009	2.868	−0.034	2.834	0.017	2.851	*Ref.*			
Female	2.881	0.044	2.925	0.074**	2.999	−0.020	2.979	0.022	0.035	0.108**	−0.037
Age											
18–35	3.007	**0.115**	3.122	−0.018	3.104	0.004	3.108	*Ref.*			
36–45	2.914	0.024	2.938	0.057	2.995	0.012	3.007	−0.093*	−0.091	0.075	0.008
46–55	2.838	0.021	2.859	0.035	2.894	−0.021	2.873	−0.169**	−0.094	0.053	0.012
56+	2.764	−0.007	2.757	−0.009	2.748	0.026	2.774	−0.243***	−0.122	0.009	−0.021
Education											
Low	2.838	0.001	2.839	0.016	2.855	−0.001	2.854	*Ref.*			
Intermediate	2.807	**0.072**	2.879	0.051	2.930	−0.013	2.917	−0.031	0.071	0.035	−0.012
High	2.815	0.032	2.847	−0.026	2.821	0.035	2.856	−0.023	0.031	−0.042	0.036
Living situation											
Alone	2.899	**0.057**	2.956	−0.028	2.928	0.016	2.944	*Ref.*			
Not alone	2.868	0.001	2.869	0.026	2.895	0.004	2.899	−0.031	−0.056	0.054	−0.012
Caring duties											
No	2.873	0.013	2.886	0.011	2.897	0.000	2.897	*Ref.*			
Yes	2.849	0.058	2.907	0.017	2.924	0.015	2.939	−0.024	0.045	0.006	0.015
Remote work											
None	2.892	0.017	2.909	0.052*	2.961	−0.011	2.950	*Ref.*			
Experienced	2.824	0.041	2.865	0.005	2.870	0.012	2.882	−0.068	0.024	−0.047	0.023
New	2.869	0.012	2.881	−0.046	2.835	0.017	2.852	−0.023	−0.005	−0.098	0.028

Note. Significant results are in bold, *p < 0.05, **p < 0.01, ***p < 0.001; µ = intercept, β = change parameter, Δ = change between groups, T = Time.

For the full sample, we observed that work engagement significantly decreased from T1 to T2 (β_*we*_ = −0.074, SE = 0.018, *p* < 0.001), increased from T2 to T3 (β_*we*_ = 0.057, SE = 0.022, *p* < 0.01), and remained stable between T3 and T4 (β_*we*_ = −0.018, SE = 0.029, *p* = 0.528). Exhaustion remained stable across T1-T2 (β_*ex*_ = 0.024, SE = 0.015, *p* = 0.118), T2-T3 (β_*ex*_ = 0.014, SE = 0.017, *p* = 0.398), and T3-T4 (β_*ex*_ = 0.002, SE = 0.022, *p* = 0.917) as none of the change scores reached statistical significance.

### Between-group comparisons

In this section, we report results on the differences in changes between groups based on sociodemographic and work characteristics of the sample.

#### Country

Employees in Switzerland reported significantly higher work engagement compared to employees in Germany at T1 (Δµ_we_ = 0.386, Δχ² (1) = 39.20, *p* < 0.001). In addition, there was a significant difference in the change between the two groups from T1 to T2 (Δβ_we_ = −0.094, Δχ² (1) = 4.51, *p* < 0.05). Work engagement decreased significantly more in Swiss employees compared to German employees. There were no significant differences in changes across T2-T3 and T3-T4. Regarding exhaustion, Swiss employees reported significantly lower exhaustion compared to German employees at T1 (Δµ_ex_ = −0.086, Δχ² (1) = 4.89, *p* < 0.05). This difference remained stable across the following measurement points as there were no significant differences in the changes across T1-T2, T2-T3, and T3-T4.

#### Gender

We did not find any significant differences in work engagement between male and female employees at T1 (Δµ_we_ = 0.108, Δχ² (1) = 3.71, *p* = 0.054). However, there was a significant difference in change from T3 to T4 (Δβ_we_ = −0.130, Δχ² (1) = 4.96, *p* < 0.05). Work engagement significantly decreased in female employees, while it remained stable in male employees. Regarding exhaustion, there were no significant differences at T1, however, there was a significant difference in change from T2 to T3 (Δβ_ex_ = 0.108, Δχ² (1) = 10.01, *p* < 0.01). Exhaustion significantly increased in female employees while we did not observe any significant changes in male employees.

#### Age

There were no significant differences in work engagement across the different age groups of employees at T1 nor any significant differences in changes across T1-T2, T2-T3, and T3-T4. However, we found significant differences in exhaustion across the four age groups of employees (Δχ² [3] = 10.01, *p* < 0.001). Overall, older employees reported significantly lower levels of exhaustion compared to younger employees at T1. Specifically, employees in the age group 36–45 years reported lower exhaustion compared to the reference group of 18–35 years (Δµ_ex_ = −0.093, *p* < 0.05); employees in the age group 46–55 years reported significantly lower exhaustion compared to the reference group of 18–35 years (Δµ_ex_ = −0.169, *p* < 0.01); and employees in the age group 56 + reported significantly lower exhaustion compared to the reference group of 18–35 years (Δµ_ex_ = −0.243, *p* < 0.001).

#### Education

There were no significant differences in work engagement or exhaustion between education groups at T1, nor were there any significant differences in changes across T1–T2, T2–T3, or T3–T4.

#### Living situation

Employees who do not live alone reported significantly higher work engagement score compared to those who live alone at T1 (Δµ = 0.253, Δχ² (1) = 15.32, *p* < 0.001). This difference remained stable across the following measurement points as there were no significant differences in changes across T1-T2, T2-T3, and T3-T4. Regarding exhaustion, there were no significant differences at T1, nor any significant differences in changes across T1-T2, T2-T3, and T3-T4.

#### Caring duties for children

Overall, employees with caring duties reported significantly higher work engagement compared to employees without caring duties at T1 (Δµ = 0.240, Δχ² (1) = 13.26, *p* < 0.001). These differences remained stable across the following measurement points as there were not any significant differences in changes across T1-T2, T2-T3, and T3-T4. Regarding exhaustion, there were no significant differences across the three groups at T1 nor any significant differences in changes across T1-T2, T2-T3, and T3-T4.

#### Remote work

Overall, experienced remote workers reported significantly higher work engagement (Δµ_we_ = 0.348, Δχ² (2) = 28.21, *p* < 0.001) compared to employees who did not work remotely at T1. No significant difference was found between employees who were new to remote work and those who did not work remotely at T1. This remained stable across the following measurement points as there were no significant differences in changes across T1-T2, T2-T3, and T3-T4. Regarding exhaustion, there were no significant differences at T1, nor any significant differences in changes across T1-T2, T2-T3, and T3-T4.

### Within-group changes

In this section, we report additional results regarding overall trends in each specific group based on sociodemographic and work characteristics of the sample.

#### Country

In German employees, work engagement significantly decreased from T1 to T2 (β_*we*_ = −0.053, SE = 0.021, *p* < 0.05) and increased from T2 to T3 (β_*we*_ = 0.052, SE = 0.024, *p* < 0.05). Similarly, in Swiss employees, work engagement significantly decreased from T1 to T2 (βµ_*we*_ = −0.147, SE = 0.039, *p* < 0.001), but remained stable thereafter. Exhaustion remained stable in both groups across the measurement period.

#### Gender

In male employees, work engagement significantly decreased from T1 to T2 (β_*we*_ = −0.062, SE = 0.024, *p* < 0.05) and increased from T2 to T3 (β_*we*_ = 0.062, SE = 0.030, *p* < 0.05). In female employees, work engagement significantly decreased from T1 to T2 (βµ_*we*_ = −0.083, SE = 0.027, *p* < 0.01) and further decreased between from T3 to T4 (β_*we*_ = −0.091, SE = 0.044, *p* < 0.05). Exhaustion remained stable in male employees, however, we observed an increase in female employees from T2 to T3 (β_*ex*_ = 0.074, SE = 0.026, *p* < 0.01).

#### Age

Work engagement significantly decreased from T1 to T2 in employees aged 46–55 (β_*we*_ = −0.073, SE = 0.034, *p* < 0.05) and 56+ (β_*we*_ = −0.133, SE = 0.030, *p* < 0.001). For the age group 56 + work engagement returned to the baseline level from T2 to T3 (β_*we*_ = 0.145, SE = 0.036, *p* < 0.001). Regarding exhaustion, there was a significant increase in the age group 18–35 from T1 to T2 (β_*ex*_ = 0.115, SE = 0.041, *p* < 0.01).

#### Education

Work engagement significantly decreased in employees with low education from T1 to T2 (β_*we*_ = −0.082, SE = 0.026, *p* < 0.01) and increased from T2 to T3 (β_*we*_ = 0.083, SE = 0.033, *p* < 0.05). Exhaustion increased from T1 to T2 in the group of employees with intermediate education (β_*ex*_ = 0.072, SE = 0.030, *p* < 0.05).

#### Living situation

Work engagement significantly decreased from T1 to T2 in employees who live alone (β_*we*_ = −0.081, SE = 0.032, *p* < 0.05) as well as in those who do not live alone (β_*we*_ = −0.057, SE = 0.022, *p* < 0.05). In employees who do not live alone, work engagement returned to the baseline from T2 to T3 (β_*we*_ = 0.056, SE = 0.026, *p* < 0.05). Exhaustion increased from T1 to T2 in the group of employees who live alone (β_*ex*_ = 0.057, SE = 0.029, *p* < 0.05).

#### Caring duties

Work engagement significantly decreased from T1 to T2 in employees without caring duties (β_*we*_ = −0.090, SE = 0.021, *p* < 0.01) as well as in those with caring duties (β_*we*_ = −0.040, SE = 0.037, *p* < 0.05). In employees without caring duties, work engagement returned to the baseline level from T2 to T3 (β_*we*_ = 0.091, SE = 0.025, *p* < 0.01). Exhaustion remained stable in both groups across the measurement period.

#### Remote work

Work engagement significantly decreased from T1 to T2 in employees who did not work remotely (β_*we*_ = −0.087, SE = 0.028, *p* < 0.01) and in those who were new to remote work (β_*we*_ = −0.077, SE = 0.037, *p* < 0.05). In both groups it returned to the baseline level from T2 to T3, however this change reached significance only in the group of employees who did not work remotely (β_*we*_ = 0.078, SE = 0.033, *p* < 0.05). Exhaustion increased from T1 to T2 in the group of employees who did not work remotely (β_*ex*_ = 0.052, SE = 0.026, *p* < 0.05).

## Discussion

The aim of our study was to examine the development of work engagement and exhaustion over time among Swiss and German employees, considering various sociodemographic and work characteristics. Our longitudinal data provided insights into how these dimensions of occupational well-being evolved from April 2020 to December 2021, encompassing significant periods of the COVID-19 pandemic.

### Overall trends

Work engagement fluctuated significantly during the COVID-19 crisis. We observed a decrease from T1 (April 2020) to T2 (June/July 2020), which coincided with the first wave of the pandemic and the initial lockdown measures introduced in late March 2020, followed by a relaxation of restrictive measures in summer 2020. This period was marked by widespread uncertainty and substantial changes in work conditions, including remote work, short-time work schemes, and layoffs, which may have contributed to lower engagement. Interestingly, work engagement then returned to baseline levels between T2 and T3 (December 2020), a period characterized by the reintroduction of more restrictive measures. Although this recovery may appear counterintuitive, one possible explanation is that employees had begun to adapt to the new work arrangements and the ongoing challenges [12, 13]. Another explanation for the earlier drop in engagement could be seasonal as the data at T2 were partially collected during the summer vacation period, when employees are more likely to psychologically detach from work. Since we did not collect data during the following summer, we were unable to empirically support this hypothesis. No further changes in work engagement were observed between T3 and T4, suggesting a possible stabilization after the initial adjustment period.

Interestingly, we did not observe any significant changes in exhaustion across the time points, which may seem surprising given the prolonged stressors of the COVID-19 pandemic. This pattern contrasts with findings from some studies that reported increased psychological distress and exhaustion during the early phases of the pandemic, particularly among healthcare workers [50, 51]. However, a meta-analysis of longitudinal studies found more heterogeneous or even stabilizing trends over time [52]. In our study, we did not differentiate between occupational groups, which may partly explain the absence of overall changes in exhaustion. The levels of exhaustion likely varied across occupations, especially with frontline workers being more affected than other occupations.

In general, most between-group differences we observed were at the baseline levels, changes over time were relatively similar across groups. This was supported by the small differences in fit indices when constraining change parameters across groups. Nonetheless, we still observed some meaningful between- and within-group differences in both, levels and trends of work engagement and exhaustion, which we discuss in detail below.

### Group differences

Swiss employees consistently reported higher work engagement and lower exhaustion compared to German employees throughout the measurement period. The overall higher levels of work engagement during the pandemic may reflect differences in national responses to the pandemic and their effects on workers as Switzerland introduced slightly less restrictive measures throughout the pandemic compared to Germany [31]. These differences may also be partially attributed to national variations in work culture, organizational practices, and broader socioeconomic conditions [53]. For instance, Switzerland tends to rank high on indicators such as job security, income levels, and general life satisfaction, which could contribute to more favorable work experiences^5^. It is therefore possible that both structural and contextual factors interacted to shape employees’ experiences during this period.

Initially, we did not observe any significant gender differences in occupational well-being. Both male and female employees experienced similar fluctuations in work engagement during the early stages of the pandemic, with a decline from T1 to T2, followed by a recovery from T2 to T3. However, we observed a significant decline in work engagement in females from T3 to T4. Moreover, female employees reported a significant increase in exhaustion from T2 to T3, which we did not observe in male employees. These findings align with previous reports [16] and may reflect overrepresentation of female employees in industries that were heavily impacted by the pandemic like human services, education, and healthcare. However, it may also reflect differences in care responsibilities, role strain, or work–nonwork boundary management. Research suggests that during the pandemic, female employees disproportionately carried the burden of caregiving and household duties, which could have contributed to elevated exhaustion and decreased capacity to recover over time [54].

Unlike previous studies, which found higher work engagement in older employees, we observed no significant age-related differences in work engagement [13]. However, older employees showed more fluctuation in work engagement, with an initial decline from T1 to T2, followed by an increase from T2 to T3. Older employees’ may have had greater initial struggles with the disruptions caused by the pandemic and required longer adaptation period. In terms of exhaustion, middle-aged and older employees (36+) reported lower exhaustion levels at T1 than younger employees [18–35]. Overall, exhaustion was lower among older participants, with the oldest 56 + age group showing the lowest levels. In particular, we observed a significant increase in exhaustion between T1 and T2 in the younger employee group [18–35] which remained constant throughout the pandemic. These findings could be attributed to factors such as career stage-specific job demands, higher job insecurity in younger employees [55], or more effective stress management by senior employees [56]. Alternatively, the trend may reflect the healthy worker effect, where healthier employees remain in the workforce longer [57].

Although the differences between groups were not significant, we observed the highest level of work engagement in highly educated employees with less fluctuation compared to employees with lower education level. These patterns may reflect more available resources and greater adaptability of those with higher education, who may have more transferrable skills, access to remote work, and greater autonomy in their job roles [16]. Interestingly, we observed a significant increase in exhaustion between T1 and T2 in the group with intermediate education. A similar, though non-significant, pattern emerged in the group with high education, whereas no such increase was found in the group with low education. Employees with higher educational level were more likely to work remotely,^6^ however, the early transition to remote work often lacked structure and was implemented under emergency conditions which may have contributed to the increase in exhaustion. Nonetheless, these results should be interpreted with caution, as the sample size for education-based group comparisons was much lower compared to other groups. Thus, the absence of additional significant differences may, in part, be attributed to missing data related to participants’ educational background.

In line with previous research [13, 24], employees living in single households reported lower work engagement compared to those living with family or friends. This highlights the importance of social support in the home environment and its potential positive influence on work motivation [58]. Additionally, a small but significant increase in exhaustion was observed from T1 to T2 in employees living alone, possibly reflecting the negative effects of social isolation during the early stages of the pandemic lockdowns [59].

Related to the benefits of social relationships, we found that employees with caring responsibilities for children reported higher work engagement across all measurement points. Our finding aligns with some existing studies showing that childcare is beneficial for employee occupational well-being [25, 60]. Perhaps caregiving roles provide additional motivation and purpose leading to positive emotions and enrichment processes in the work domain [55]. Despite these positive effects, no significant differences were observed in work-related exhaustion between employees with and without caring duties. Since our quantitative measure focused specifically on work-related exhaustion, it might not fully capture the broader impact of caregiving duties on employee energy levels.

Finally, in line with previous studies [12, 24, 25], we found that remote work was associated with higher work engagement. However, we observed this only in employees with prior remote work experience who reported significantly higher work engagement compared to those who had no remote work experience or who began remote work during the pandemic. This suggests that prior remote work experience helps employees better navigate the challenges of remote work, such as managing work-home boundaries, loneliness, or self-discipline [61]. Additionally, we observed more fluctuations in both work engagement and exhaustion in employees who did not work remotely. Unlike remote workers, these employees were possibly more directly affected by changing government regulations and workplace safety protocols, which may have disrupted their routines and increased stress levels over time. Accordingly, the increase in exhaustion at T3 may suggest that on-site work during renewed restrictive measures in late 2020 may have posed additional psychological demands. Our findings emphasize the benefits of remote work but also highlight that prior experience appears to be crucial for this work arrangement during the times of crises [59].

### Strengths and limitations

The main strength of this study is a large sample size, which enabled comprehensive group analyses and increased generalizability of findings across different groups of employees. Additionally, the study’s longitudinal design, including four waves of measurement, provided a robust framework for capturing changes over time during the dynamic conditions of the COVID-19 pandemic. Finally, the use of LCSM is another important strength, which significantly reduced the risk of the confounding effects of measurement error and enhanced the reliability of our findings.

However, there are several limitations to consider. First, relying on self-reported data may introduce biases, such as social desirability or recall bias [62]. We aimed to mitigate these through careful survey design and anonymity assurances [63]. Second, missing data are a common challenge in longitudinal research due to participant attrition. Although the attrition analysis identified some marginal but significant differences in terms of age, gender, and country, we did not observe any significant differences in the indicators of occupational well-being. In addition, we used full information maximum likelihood estimation, which allowed us to incorporate the observed patterns of missingness into our analyses to mitigate potential biases introduced by participant dropout across waves [64]. Another related limitation is that we did not consider participants who reported changes in sociodemographic characteristics or selected the response option “other.” In our study, however, the number of excluded cases was very small (less than 1%), making it unlikely that this affected the results. For future studies with larger datasets, it would be worthwhile to account for such cases by treating sociodemographic variables as time-varying covariates. Third, the generalizability of our findings is limited by the sample composition, which primarily included professionals from Western countries. Although we used quota sampling to approximate the general working population, the sample cannot be considered fully representative. Gender distribution matches the general population well, but our participants tend to be older. Moreover, we did not differentiate between occupational sectors which limits our ability to assess how specific job characteristics, such as those found in healthcare, education, or manual labor, may have differently influenced work engagement and exhaustion during the pandemic. In addition,the study was conducted predominantly in the context of the COVID-19 without pre-pandemic data which limits the possibility to fully attribute observed changes in occupational well-being to pandemic-related factors alone. Other contextual influences (e.g., seasonality or holiday effects) cannot be entirely ruled out. Finally, we were able to establish only partial measurement invariance for exhaustion and, in case of a few groups, also for work engagement. These contextual and methodological limitations highlight the need for cautious interpretation of our findings and consideration of broader contextual factors influencing occupational well-being during the study period.

## Conclusion

Our study provides interesting insights into the dynamic and group-specific nature of occupational well-being during the COVID-19 pandemic. Work engagement showed temporal fluctuations, with an initial decline followed by partial recovery and stabilization, highlighting the importance of adaptation to crises. Social connections emerged as a critical factor, as employees living with others and those with caregiving responsibilities reported higher work engagement, emphasizing the motivational role of interpersonal relationships. Group differences further underscore the role of national context and work flexibility as Swiss employees and those with prior remote work experience consistently reported higher work engagement. While exhaustion levels remained stable overall, we identified some important country- and age-specific differences, potentially reflecting differences in national responses to the pandemic and the strain experienced particularly by younger employees. We encourage future studies focusing on diverse cultural contexts with longitudinal designs to enhance insights into the interplay of sociodemographic factors, work characteristics, and occupational well-being.

## Supplementary Information

Supplementary Material 1.

Supplementary Material 2.

Supplementary Material 3.

## Data Availability

The datasets used and/or analysed during the current study are available from the corresponding author upon reasonable request. The R code used for the statistical analysis is available as a supplementary material.

## Abbreviations

JD-R: Job Demands-Resources
ISCED: International Standard Classification of Education
CFA: Confirmatory Factor Analysis
RMSEA: Root Mean Square Error of Approximation
CFI: Comparative Fit Index
TLI: Tucker-Lewis Index
SRMR: Standardized Root Mean Square Residual
LCSM: Latent Change Score Modeling

## Footnotes

1
https://data.snf.ch/grants/grant/201113 [cited 2025 Sep 30].

3 Table S1 and Table S2 in Supplementary Material 3.

4 Table S4 in Supplementary Material 3.

5 Federal Statistical Office (FSO). Statistics on income and living conditions (SILC) [Internet]. Neuchâtel: Federal Statistical Office; [cited 2025 Jul 16]. Available from: https://www.bfs.admin.ch/bfs/en/home/statistiken/wirtschaftliche-soziale-situation-bevoelkerung/wohlbefinden-armut/subjektives-wohlbefinden-lebensbedingungen/subjektives-wohlbefinden-schweiz-europa.html.

6 In our sample, 64% of employees with a high level of education, 48% with an intermediate level, and 37% with a low level worked remotely.
