# Supplementary material for: Longitudinal changes in occupational well-being: a four-wave panel survey of employees in Switzerland and Germany
Source: BMC Public Health. 2025 Nov 28;25:4187. doi: 10.1186/s12889-025-25237-z (PMC12664253; doi:10.1186/s12889-025-25237-z)
Supplement: Supplementary file 1 — Supplementary Material 1. [file 12889_2025_25237_MOESM1_ESM.pdf]

Example R code_LCSM & plots for work engagement

Masked

2024-10-11

```
### Latent Change Score Model -----------
## Work engagement ----------------
# Latent mean freely estimated
model1a <- '
  # Specify latent true scores
  WE.1 =~ s32_vi.4 + a2*s32_de.4 + a3*s32_ab.4
  WE.2 =~ s32_vi.5 + a2*s32_de.5 + a3*s32_ab.5
  WE.3 =~ s32_vi.6 + a2*s32_de.6 + a3*s32_ab.6
  WE.4 =~ s32_vi.7 + a2*s32_de.7 + a3*s32_ab.7

  # Specify manifest intercepts
  s32_de.4 ~ a4*1
  s32_de.5 ~ a4*1
  s32_de.6 ~ a4*1
  s32_de.7 ~ a4*1
  s32_ab.4 ~ a5*1
  s32_ab.5 ~ a5*1
  s32_ab.6 ~ a5*1
  s32_ab.7 ~ a5*1

  # Residual covariances between same items
  s32_vi.4 ~~ s32_vi.5 + s32_vi.6 + s32_vi.7
  s32_vi.5 ~~ s32_vi.6 + s32_vi.7
  s32_vi.6 ~~ s32_vi.7
  s32_de.4 ~~ s32_de.5 + s32_de.6 + s32_de.7
  s32_de.5 ~~ s32_de.6 + s32_de.7
  s32_de.6 ~~ s32_de.7
  s32_ab.4 ~~ s32_ab.5 + s32_ab.6 + s32_ab.7
  s32_ab.5 ~~ s32_ab.6 + s32_ab.7
  s32_ab.6 ~~ s32_ab.7

  # Specify mean of latent true scores
  WE.1 ~ 1
  WE.2 ~ 0*1
  WE.3 ~ 0*1
  WE.4 ~ 0*1

  # Specify variance of latent true scores
  WE.1 ~~ WE.1
  WE.2 ~~ 0*WE.2
  WE.3 ~~ 0*WE.3
  WE.4 ~~ 0*WE.4
```

```r
  # Specify autoregressions of latent variables
  WE.2 ~ 1*WE.1
  WE.3 ~ 1*WE.2
  WE.4 ~ 1*WE.3

  # Specify latent change scores
  d2 =~ 1*WE.2
  d3 =~ 1*WE.3
  d4 =~ 1*WE.4

  # Specify latent change scores means
  d2 ~ 1
  d3 ~ 1
  d4 ~ 1

  # Specify latent change scores variances
  d2 ~~ d2
  d3 ~~ d3
  d4 ~~ d4

  # Intercepts of reference variables set to zero
  s32_vi.4 ~ 0*1
  s32_vi.5 ~ 0*1
  s32_vi.6 ~ 0*1
  s32_vi.7 ~ 0*1

  # Change factors covariances
  d2 ~~ d3 + d4
  d3 ~~ d4
'

# Latent mean fixed to be equal
model1b <- '
  # Specify latent true scores
  WE.1 =~ s32_vi.4 + a2*s32_de.4 + a3*s32_ab.4
  WE.2 =~ s32_vi.5 + a2*s32_de.5 + a3*s32_ab.5
  WE.3 =~ s32_vi.6 + a2*s32_de.6 + a3*s32_ab.6
  WE.4 =~ s32_vi.7 + a2*s32_de.7 + a3*s32_ab.7

  # Specify manifest intercepts
  s32_de.4 ~ a4*1
  s32_de.5 ~ a4*1
  s32_de.6 ~ a4*1
  s32_de.7 ~ a4*1
  s32_ab.4 ~ a5*1
  s32_ab.5 ~ a5*1
  s32_ab.6 ~ a5*1
  s32_ab.7 ~ a5*1

  # Residual covariances between same items
  s32_vi.4 ~~ s32_vi.5 + s32_vi.6 + s32_vi.7
  s32_vi.5 ~~ s32_vi.6 + s32_vi.7
  s32_vi.6 ~~ s32_vi.7
```

```
  s32_de.4 ~~ s32_de.5 + s32_de.6 + s32_de.7
  s32_de.5 ~~ s32_de.6 + s32_de.7
  s32_de.6 ~~ s32_de.7
  s32_ab.4 ~~ s32_ab.5 + s32_ab.6 + s32_ab.7
  s32_ab.5 ~~ s32_ab.6 + s32_ab.7
  s32_ab.6 ~~ s32_ab.7

  # Specify mean of latent true scores
  WE.1 ~ i*1
  WE.2 ~ 0*1
  WE.3 ~ 0*1
  WE.4 ~ 0*1

  # Specify variance of latent true scores
  WE.1 ~~ WE.1
  WE.2 ~~ 0*WE.2
  WE.3 ~~ 0*WE.3
  WE.4 ~~ 0*WE.4

  # Specify autoregressions of latent variables
  WE.2 ~ 1*WE.1
  WE.3 ~ 1*WE.2
  WE.4 ~ 1*WE.3

  # Specify latent change scores
  d2 =~ 1*WE.2
  d3 =~ 1*WE.3
  d4 =~ 1*WE.4

  # Specify latent change scores means
  d2 ~ 1
  d3 ~ 1
  d4 ~ 1

  # Specify latent change scores variances
  d2 ~~ d2
  d3 ~~ d3
  d4 ~~ d4

  # Intercepts of reference variables set to zero
  s32_vi.4 ~ 0*1
  s32_vi.5 ~ 0*1
  s32_vi.6 ~ 0*1
  s32_vi.7 ~ 0*1

  # Change factors covariances
  d2 ~~ d3 + d4
  d3 ~~ d4
'

# Latent change score d2 fixed to be equal
model1c <- '
  # Specify latent true scores
```

```
WE.1 =~ s32_vi.4 + a2*s32_de.4 + a3*s32_ab.4
WE.2 =~ s32_vi.5 + a2*s32_de.5 + a3*s32_ab.5
WE.3 =~ s32_vi.6 + a2*s32_de.6 + a3*s32_ab.6
WE.4 =~ s32_vi.7 + a2*s32_de.7 + a3*s32_ab.7

# Specify manifest intercepts
s32_de.4 ~ a4*1
s32_de.5 ~ a4*1
s32_de.6 ~ a4*1
s32_de.7 ~ a4*1
s32_ab.4 ~ a5*1
s32_ab.5 ~ a5*1
s32_ab.6 ~ a5*1
s32_ab.7 ~ a5*1

# Residual covariances between same items
s32_vi.4 ~~ s32_vi.5 + s32_vi.6 + s32_vi.7
s32_vi.5 ~~ s32_vi.6 + s32_vi.7
s32_vi.6 ~~ s32_vi.7
s32_de.4 ~~ s32_de.5 + s32_de.6 + s32_de.7
s32_de.5 ~~ s32_de.6 + s32_de.7
s32_de.6 ~~ s32_de.7
s32_ab.4 ~~ s32_ab.5 + s32_ab.6 + s32_ab.7
s32_ab.5 ~~ s32_ab.6 + s32_ab.7
s32_ab.6 ~~ s32_ab.7

# Specify mean of latent true scores
WE.1 ~ 1
WE.2 ~ 0*1
WE.3 ~ 0*1
WE.4 ~ 0*1

# Specify variance of latent true scores
WE.1 ~~ WE.1
WE.2 ~~ 0*WE.2
WE.3 ~~ 0*WE.3
WE.4 ~~ 0*WE.4

# Specify autoregressions of latent variables
WE.2 ~ 1*WE.1
WE.3 ~ 1*WE.2
WE.4 ~ 1*WE.3

# Specify latent change scores
d2 =~ 1*WE.2
d3 =~ 1*WE.3
d4 =~ 1*WE.4

# Specify latent change scores means
d2 ~ i*1
d3 ~ 1
d4 ~ 1
```

```
  # Specify latent change scores variances
  d2 ~~ d2
  d3 ~~ d3
  d4 ~~ d4

  # Intercepts of reference variables set to zero
  s32_vi.4 ~ 0*1
  s32_vi.5 ~ 0*1
  s32_vi.6 ~ 0*1
  s32_vi.7 ~ 0*1

  # Change factors covariances
  d2 ~~ d3 + d4
  d3 ~~ d4
'

# Latent change score d3 fixed to be equal
model1d <- '
  # Specify latent true scores
  WE.1 =~ s32_vi.4 + a2*s32_de.4 + a3*s32_ab.4
  WE.2 =~ s32_vi.5 + a2*s32_de.5 + a3*s32_ab.5
  WE.3 =~ s32_vi.6 + a2*s32_de.6 + a3*s32_ab.6
  WE.4 =~ s32_vi.7 + a2*s32_de.7 + a3*s32_ab.7

  # Specify manifest intercepts
  s32_de.4 ~ a4*1
  s32_de.5 ~ a4*1
  s32_de.6 ~ a4*1
  s32_de.7 ~ a4*1
  s32_ab.4 ~ a5*1
  s32_ab.5 ~ a5*1
  s32_ab.6 ~ a5*1
  s32_ab.7 ~ a5*1

  # Residual covariances between same items
  s32_vi.4 ~~ s32_vi.5 + s32_vi.6 + s32_vi.7
  s32_vi.5 ~~ s32_vi.6 + s32_vi.7
  s32_vi.6 ~~ s32_vi.7
  s32_de.4 ~~ s32_de.5 + s32_de.6 + s32_de.7
  s32_de.5 ~~ s32_de.6 + s32_de.7
  s32_de.6 ~~ s32_de.7
  s32_ab.4 ~~ s32_ab.5 + s32_ab.6 + s32_ab.7
  s32_ab.5 ~~ s32_ab.6 + s32_ab.7
  s32_ab.6 ~~ s32_ab.7

  # Specify mean of latent true scores
  WE.1 ~ 1
  WE.2 ~ 0*1
  WE.3 ~ 0*1
  WE.4 ~ 0*1

  # Specify variance of latent true scores
  WE.1 ~~ WE.1
```

```
  WE.2 ~~ 0*WE.2
  WE.3 ~~ 0*WE.3
  WE.4 ~~ 0*WE.4

  # Specify autoregressions of latent variables
  WE.2 ~ 1*WE.1
  WE.3 ~ 1*WE.2
  WE.4 ~ 1*WE.3

  # Specify latent change scores
  d2 =~ 1*WE.2
  d3 =~ 1*WE.3
  d4 =~ 1*WE.4

  # Specify latent change scores means
  d2 ~ 1
  d3 ~ i*1
  d4 ~ 1

  # Specify latent change scores variances
  d2 ~~ d2
  d3 ~~ d3
  d4 ~~ d4

  # Intercepts of reference variables set to zero
  s32_vi.4 ~ 0*1
  s32_vi.5 ~ 0*1
  s32_vi.6 ~ 0*1
  s32_vi.7 ~ 0*1

  # Change factors covariances
  d2 ~~ d3 + d4
  d3 ~~ d4
'

# Latent change score d4 fixed to be equal
model1e <- '
  # Specify latent true scores
  WE.1 =~ s32_vi.4 + a2*s32_de.4 + a3*s32_ab.4
  WE.2 =~ s32_vi.5 + a2*s32_de.5 + a3*s32_ab.5
  WE.3 =~ s32_vi.6 + a2*s32_de.6 + a3*s32_ab.6
  WE.4 =~ s32_vi.7 + a2*s32_de.7 + a3*s32_ab.7

  # Specify manifest intercepts
  s32_de.4 ~ a4*1
  s32_de.5 ~ a4*1
  s32_de.6 ~ a4*1
  s32_de.7 ~ a4*1
  s32_ab.4 ~ a5*1
  s32_ab.5 ~ a5*1
  s32_ab.6 ~ a5*1
  s32_ab.7 ~ a5*1
```

```
  # Residual covariances between same items
  s32_vi.4 ~~ s32_vi.5 + s32_vi.6 + s32_vi.7
  s32_vi.5 ~~ s32_vi.6 + s32_vi.7
  s32_vi.6 ~~ s32_vi.7
  s32_de.4 ~~ s32_de.5 + s32_de.6 + s32_de.7
  s32_de.5 ~~ s32_de.6 + s32_de.7
  s32_de.6 ~~ s32_de.7
  s32_ab.4 ~~ s32_ab.5 + s32_ab.6 + s32_ab.7
  s32_ab.5 ~~ s32_ab.6 + s32_ab.7
  s32_ab.6 ~~ s32_ab.7

  # Specify mean of latent true scores
  WE.1 ~ 1
  WE.2 ~ 0*1
  WE.3 ~ 0*1
  WE.4 ~ 0*1

  # Specify variance of latent true scores
  WE.1 ~~ WE.1
  WE.2 ~~ 0*WE.2
  WE.3 ~~ 0*WE.3
  WE.4 ~~ 0*WE.4

  # Specify autoregressions of latent variables
  WE.2 ~ 1*WE.1
  WE.3 ~ 1*WE.2
  WE.4 ~ 1*WE.3

  # Specify latent change scores
  d2 =~ 1*WE.2
  d3 =~ 1*WE.3
  d4 =~ 1*WE.4

  # Specify latent change scores means
  d2 ~ 1
  d3 ~ 1
  d4 ~ i*1

  # Specify latent change scores variances
  d2 ~~ d2
  d3 ~~ d3
  d4 ~~ d4

  # Intercepts of reference variables set to zero
  s32_vi.4 ~ 0*1
  s32_vi.5 ~ 0*1
  s32_vi.6 ~ 0*1
  s32_vi.7 ~ 0*1

  # Change factors covariances
  d2 ~~ d3 + d4
  d3 ~~ d4
'
```

```r
# Groups: gender, age, edu, liv, ho, country, care
fit1a <- sem(model1a,
           data = df,
           estimator = "ML",
           missing = "FIML",
           group = "gender")

summary(fit1a,
        rsquare = TRUE,
        standardized = TRUE,
        fit.measures = TRUE)

fitMeasures(fit1a, c("chisq", "pvalue", "df", "cfi", "tli", "rmsea","srmr"))

fit1b <- sem(model1b,
           data = df,
           estimator = "ML",
           missing = "FIML",
           group = "gender")

summary(fit1b,
        rsquare = TRUE,
        standardized = TRUE,
        fit.measures = TRUE)

fitMeasures(fit1b, c("chisq", "pvalue", "df", "cfi", "tli", "rmsea","srmr"))

anova(fit1a, fit1b)

fit1c <- sem(model1c,
             data = df,
             estimator = "ML",
             missing = "FIML",
             group = "liv_ho")

summary(fit1c,
        rsquare = TRUE,
        standardized = TRUE,
        fit.measures = TRUE)

fitMeasures(fit1c, c("chisq", "pvalue", "df", "cfi", "tli", "rmsea","srmr"))

anova(fit1a, fit1c)

fit1d <- sem(model1d,
             data = df,
             estimator = "ML",
             missing = "FIML",
             group = "liv_ho")

summary(fit1d,
        rsquare = TRUE,
        standardized = TRUE,
```

```
        fit.measures = TRUE)

fitMeasures(fit1d, c("chisq", "pvalue", "df", "cfi", "tli", "rmsea","srmr"))

anova(fit1a, fit1d)

fit1e <- sem(model1e,
             data = df,
             estimator = "ML",
             missing = "FIML",
             group = "liv_ho")

summary(fit1e,
        rsquare = TRUE,
        standardized = TRUE,
        fit.measures = TRUE)

fitMeasures(fit1e, c("chisq", "pvalue", "df", "cfi", "tli", "rmsea","srmr"))

anova(fit1a, fit1e)

### Plots: example code -----------------
## Full sample
fit1a <- sem(model1a,
             data = df,
             estimator = "ML",
             missing = "FIML")

tmp <- parameterEstimates(fit1a, standardized = TRUE, remove.nonfree = TRUE)
tmp$param <- paste(tmp$lhs, tmp$op, tmp$rhs, sep = " ")

tabledata <- tmp
tabledata <- filter(tmp, param %in% c("WE.1 ~1 ", "d2 ~1 ", "d3 ~1 ", "d4 ~1 "))

tabledata$mean <- tabledata$est

tabledata$mean[tabledata$param == "d2 ~1 "] <- tabledata$est[tabledata$param == "WE.1 ~1 "] + # Interce
  tabledata$est[tabledata$param == "d2 ~1 "] # Change Score

tabledata$mean[tabledata$param == "d3 ~1 "] <- tabledata$mean[tabledata$param == "d2 ~1 "] + # Intercep
  tabledata$est[tabledata$param == "d3 ~1 "] # Change Score

tabledata$mean[tabledata$param == "d4 ~1 "] <- tabledata$mean[tabledata$param == "d3 ~1 "] + # Intercep
  tabledata$est[tabledata$param == "d4 ~1 "] # Change Score

plotdata <- filter(tabledata, param %in% c("WE.1 ~1 ", "d2 ~1 ", "d3 ~1 ", "d4 ~1 "))

plotdata <- plotdata %>%
  mutate(wave = case_when(
    param %in% c("WE.1 ~1 ") ~ (ym("2020-04")),
    param %in% c("d2 ~1 ") ~ ym("2020-07"),
    param %in% c("d3 ~1 ") ~ ym("2020-12"),
    param %in% c("d4 ~1 ") ~ ym("2021-12")),
```

```
    outcome = case_when(
      param %in% c("WE.1 ~1 ", "d2 ~1 ", "d3 ~1 ", "d4 ~1 ") ~ "Work engagement"),
    )

p1 <- ggplot(plotdata, mapping = aes(x = wave, y = mean, group = outcome)) +
  geom_line(show.legend = FALSE) +
  geom_point(mapping = aes(shape = outcome), show.legend = FALSE) +
  theme_apa() +
  ylim(2, 4) +
  xlab(NULL) +
  ylab(NULL) +
  ggtitle("Full sample") +
  scale_x_date(date_labels = "%b %Y", breaks = as.Date(c("2020-04-01", "2020-07-01", "2020-12-01", "202[illegible]
p1

## Gender - male, female
fit1a <- sem(model1a,
             data = df,
             estimator = "ML",
             missing = "FIML",
             group = "gender")

tmp <- parameterEstimates(fit1a, standardized = TRUE, remove.nonfree = TRUE)
tmp$param <- paste(tmp$lhs, tmp$op, tmp$rhs, sep = " ")

tabledata <- tmp
tabledata <- filter(tmp, param %in% c("WE.1 ~1 ", "d2 ~1 ", "d3 ~1 ", "d4 ~1 "))

tabledata$group <- factor(tabledata$group, levels = c(1:2), labels = c("Male", "Female"))

tabledata$mean <- tabledata$est

# Male
tabledata$mean[tabledata$param == "d2 ~1 " & tabledata$group == "Male"] <- tabledata$est[tabledata$para[illegible]
  tabledata$est[tabledata$param == "d2 ~1 " & tabledata$group == "Male"] # Change Score

tabledata$mean[tabledata$param == "d3 ~1 " & tabledata$group == "Male"] <- tabledata$mean[tabledata$para[illegible]
  tabledata$est[tabledata$param == "d3 ~1 " & tabledata$group == "Male"] # Change Score

tabledata$mean[tabledata$param == "d4 ~1 " & tabledata$group == "Male"] <- tabledata$mean[tabledata$para[illegible]
  tabledata$est[tabledata$param == "d4 ~1 " & tabledata$group == "Male"] # Change Score

# Female
tabledata$mean[tabledata$param == "d2 ~1 " & tabledata$group == "Female"] <- tabledata$est[tabledata$pa[illegible]
  tabledata$est[tabledata$param == "d2 ~1 " & tabledata$group == "Female"] # Change Score

tabledata$mean[tabledata$param == "d3 ~1 " & tabledata$group == "Female"] <- tabledata$mean[tabledata$pa[illegible]
  tabledata$est[tabledata$param == "d3 ~1 " & tabledata$group == "Female"] # Change Score

tabledata$mean[tabledata$param == "d4 ~1 " & tabledata$group == "Female"] <- tabledata$mean[tabledata$pa[illegible]
  tabledata$est[tabledata$param == "d4 ~1 " & tabledata$group == "Female"] # Change Score

plotdata <- filter(tabledata, param %in% c("WE.1 ~1 ", "d2 ~1 ", "d3 ~1 ", "d4 ~1 "))
```

```
plotdata <- plotdata %>%
  mutate(wave = case_when(
    param %in% c("WE.1 ~1 ") ~ (ym("2020-04")),
    param %in% c("d2 ~1 ") ~ ym("2020-07"),
    param %in% c("d3 ~1 ") ~ ym("2020-12"),
    param %in% c("d4 ~1 ") ~ ym("2021-12")),
    outcome = case_when(
      param %in% c("WE.1 ~1 ", "d2 ~1 ", "d3 ~1 ", "d4 ~1 ") ~ "Work engagement"),
  )

plotdata$group <- factor(plotdata$group, levels = c("Female", "Male"))

p2 <- ggplot(plotdata, mapping = aes(x = wave, y = mean)) +
  geom_point(mapping = aes(shape = group), show.legend = TRUE) +
  geom_line(mapping = aes(linetype = group), show.legend = TRUE) +
  theme_apa() +
  ylim(1.5, 4.5) +
  xlab(NULL) +
  ylab(NULL) +
  ggtitle("Gender") +
  scale_x_date(date_labels = "%b %Y", breaks = as.Date(c("2020-04-01", "2020-07-01", "2020-12-01", "202
```
