# Supplementary material for: Longitudinal changes in occupational well-being: a four-wave panel survey of employees in Switzerland and Germany
Source: BMC Public Health. 2025 Nov 28;25:4187. doi: 10.1186/s12889-025-25237-z (PMC12664253; doi:10.1186/s12889-025-25237-z)
Supplement: Supplementary file 2 — Supplementary Material 2. [file 12889_2025_25237_MOESM2_ESM.pdf]

Longitudinal Changes in Occupational Well-Being: A Four-Wave Panel Survey of Employees in Switzerland and Germany

Martin Tušl[1*], Anja I. Lehmann[1], Anja I. Morstatt[1, 2], Holger Dressel[3], Georg F. Bauer[1]

[1] Public and Organizational Health, Center of Salutogenesis, Institute of Epidemiology, Biostatistics, and Prevention, University of Zurich, Zurich, Switzerland

[2] Work, Organizational and Social Psychology, Institute of Psychology, Technical University of Braunschweig, 38106 Braunschweig, Germany

[3] Occupational and Environmental Medicine, Institute of Epidemiology, Biostatistics, and Prevention, University of Zurich, Zurich, Switzerland

*corresponding author, email: martin.tusl@uzh.ch

Instructions for completing the questionnaire:

- Answer the questions spontaneously, as it applies to you at the moment.
- There are no right or wrong answers. It is exclusively about your personal opinion and experience.

Anonymity

- All information will be treated strictly confidentially. The data will be analyzed at the University of Zurich.
- The results will be reported completely anonymously, i.e. only in the form of average values or percentages.

Thank you very much for your participation!

Are you employed?
( ) Yes
( ) No

How many hours per week do you work as part of your employment contract?
( ) 0-9 hours
( ) 10-19 hours
( ) 20-29 hours
( ) 30-39 hours
( ) 40-49 hours
( ) more than 49 hours

Year of birth
(select)

Your gender
( ) Male
( ) Female
( ) Other / Do not want to answer

What is the highest level of education you have completed with a certificate or diploma?
( ) No degree **(low)**
( ) Secondary school diploma / Compulsory education **(low)**
( ) Apprenticeship, vocational school, vocational baccalaureate school **(low)**
( ) High school diploma **(intermediate)**
( ) University of applied sciences **(intermediate)**
( ) University, higher education **(high)**

Country you live in
( ) Germany
( ) Switzerland
( ) Other

What is your current living situation?
( ) I live alone
( ) I live with my partner / family
( ) I live in a shared household
( ) Other

Do you have children under 18 years who live in your household and require your care?
( ) No
( ) Yes

The share of my work that I perform in the home office was/is as follows:
before the Corona crisis (in percent) 0%-100%
since the Corona crisis (in percent) 0%-100%

Work engagement (Schaufeli et al., 2006)
The following statements are about how you feel at work. Please read each statement carefully and decide if you ever feel this way about your job. If you have never had this feeling, cross the '1' in the space after the statement. If you have had this feeling, indicate how often you feel it by crossing the number (from 2 to 7) that best describes how frequently you feel that way.

(0) Never, (1) Almost never, (2) Rarely, (3) Sometimes, (4) Often, (5) Very often, (6) Always

1. At my work, I feel bursting with energy (*vigor*)
2. At my job, I feel strong and vigorous (*vigor*)
3. I am enthusiastic about my job (*dedication*)
4. My job inspires me (*dedication*)
5. When I get up in the morning, I feel like going to work (*vigor*)
6. I feel happy when I am working intensely (*absorption*)
7. I am proud on the work that I do (*dedication*)
8. I am immersed in my work (*absorption*)
9. I get carried away when I'm working (*absorption*)

Work-related burnout (Kristensen et al., 2005; items used in the study are in **bold**)
Please read each statement carefully and indicate the degree to which you experience these feelings in your daily life. There are no right or wrong answers; simply respond as honestly as possible based on your personal experience.

(1) Never/Almost never, (2) Rarely, (3) Sometimes, (4) Often, (5) Very often

1. **Do you feel worn out at the end of the working day?**
2. **Are you exhausted in the morning at the thought of another day at work?**
3. **Do you feel that every working hour is tiring for you?**
4. Do you have enough energy for family and friends during leisure time?

(1) To a very low degree, (2) To a low degree, (3) Somewhat, (4) To a high degree, (5) To a very high degree

5. Is your work emotionally exhausting?
6. Does your work frustrate you?
7. Do you feel burnt out because of your work?
